# Highly Fast Response of Pd/Ta_2_O_5_/SiC and Pd/Ta_2_O_5_/Si Schottky Diode-Based Hydrogen Sensors

**DOI:** 10.3390/s21041042

**Published:** 2021-02-03

**Authors:** Muhammad Hussain, Woonyoung Jeong, Il-Suk Kang, Kyeong-Keun Choi, Syed Hassan Abbas Jaffery, Asif Ali, Tassawar Hussain, Muhammad Ayaz, Sajjad Hussain, Jongwan Jung

**Affiliations:** 1 Department of Nanotechnology and Advanced Materials Engineering and HMC, Sejong University, Seoul 05006, Korea; mhussainsju@gmail.com (M.H.); raehyang2@naver.com (W.J.); hassan@sju.ac.kr (S.H.A.J.); asifmju@gmail.com (A.A.); tassawar517i@gmail.com (T.H.); hussain@sejong.ac.kr (S.H.); 2 National Nanofab Center, Korea Advanced Institute of Science and Technology, 291 Daehak-ro, Yuseong-gu, Daejeon 34141, Korea; iskang@nnfc.re.kr; 3 NINT (National Institute for Nanomaterials Technology), Pohang University of Science and Technology, Pohang 37673, Korea; choikk@postech.ac.kr; 4 Department of Electronic Engineering, Semyung University, Chungcheongbuk-do 27136, Korea; 2017135801@semyung.ac.kr

**Keywords:** hydron sensor, high temperature, Schottky diode, tantalum oxide, silicon carbide

## Abstract

Herein, the fabrication of a novel highly sensitive and fast hydrogen (H_2_) gas sensor, based on the Ta_2_O_5_ Schottky diode, is described. First, Ta_2_O_5_ thin films are deposited on silicon carbide (SiC) and silicon (Si) substrates via a radio frequency (RF) sputtering method. Then, Pd and Ni are respectively deposited on the front and back of the device. The deposited Pd serves as a H_2_ catalyst, while the Ni functions as an Ohmic contact. The devices are then tested under various concentrations of H_2_ gas at operating temperatures of 300, 500, and 700 °C. The results indicate that the Pd/Ta_2_O_5_ Schottky diode on the SiC substrate exhibits larger concentration and temperature sensitivities than those of the device based on the Si substrate. In addition, the optimum operating temperature of the Pd/Ta_2_O_5_ Schottky diode for use in H_2_ sensing is shown to be about 300 °C. At this optimum temperature, the dynamic responses of the sensors towards various concentrations of H_2_ gas are then examined under a constant bias current of 1 mA. The results indicate a fast rise time of 7.1 s, and a decay of 18 s, for the sensor based on the SiC substrate.

## 1. Introduction

Hydrogen (H_2_) gas detection is widely used in various fields such as environmental monitoring, domestic/industrial safety control, seismic surveillance, semiconductor technology, and chemical/industrial process control (e.g., in nuclear reactors and coal mines). However, the colorless, odorless, and toxic nature of hydrogen gas renders it undetectable by the human eyes or nose, while its readily diffusive, highly flammable, corrosive, and explosive nature can result in disastrous consequences in the event of a leakage. Therefore, the development of a simple and efficient H_2_ sensor with high sensitivity, selectivity, and reliability, along with a rapid response and wide working temperature range, remains a significant challenge.

Numerous approaches have been adopted to design and develop hydrogen-based sensors with various structures and mechanisms, including hollow architectures such as spheres [1,2], nanowires/nanofibers [3,4], nanorods [5], hemispheres [6], and inorganic tubes [7]. For improved sensing performance, various synthetic methods have been used to control the morphology and structure so as to provide a high surface-to-volume ratio, easy electron/ion diffusion, large penetration depth, and fast response/recovery times [8]. Various semiconducting metal oxides have been used in the fabrication of resistive H_2_ sensor applications, including n-type (TiO_2_, WO_3_, ZrO_2_, CeO_2_, In_2_O_3_, Nb_2_O_5_, ZnO and SnO_2_) [9,10,11,12,13,14,15], p-type (CuO, CoO, Co_3_O_4_ and NiO) [16,17,18,19], or multilayered metal oxides decorated with noble metal catalysts such as gold (Au), platinum (Pt), ruthenium (Ru), or palladium (Pd) based compounds such as Pd/WO_3_, Pd/V_2_O_5_, Pd/SnO_2_, and Pd/ZnO. Although these precious metal catalysts exhibit superior room-temperature hydrogen solubility, many metal oxides or semiconductors are not commercially viable due to excessive power consumption, instability, poor selectivity, sensitivity, and accuracy at high operating temperatures. Moreover, a significant drawback in the use of Pd metal as a highly-sensitive hydrogen detector is the strong tendency for its electrical resistance to exhibit hysteresis due to the facile adsorption of hydrogen into the Pd structure [20,21]. In view of these challenges, recent research is focusing on the replacement of metal oxide-based materials to achieve high sensitivity and selectivity along with a higher response under a range of working H_2_ concentrations, as well as improved cost effectiveness and reproducibility.

Recently, two-dimensional (2D) materials have attracted much interest due to their unique structural and extraordinary sensing properties [22,23]. In addition, previous studies have reported that doping and creating defects are two effective methods for enhancing the sensing or catalytic performance of 2D materials [24,25]. For example, much progress has been made in the development of sensors for NH_3_, SO_2_, NO_2_, and CO_2_ gases based on layered transition metal dichalcogenides (LTMDs) such as GeSe [26], graphene and reduced graphene oxide (RGO) [27,28], phosphorene [29], MoS_2_ [30], WS_2_ [31,32], and vanadium carbide MXene (V_2_CTx) [33]. However, while the research into 2D materials for H_2_-based sensors has produced significant success, the study is still in its infancy and few reports have been published. Moreover, the attainment of a satisfactory performance remains hampered by the complicated fabrication process, insufficient hydrogen sensitivity/detection, lower response time, and shorter desorption time.

In addition, while bare Si cannot meet the requirements for application in severe environments, the three-times wider bandgap of the group-III nitride silicon carbide (SiC) (3.26 eV) enables operation at temperatures of up to 1000 °C along with an excellent thermal conductivity of 3.0–4.9 W/cm K. Moreover, the high stability of the carbon/silicon bonding, the low diffusion coefficient, and the critical electric field over eight times that of silicon, enable the production of SiC-based electronic devices with low power loss, thus potentially increasing the battery life [34]. Furthermore, the ease of fabrication of the SiC-based gas sensors on a smaller chip, to provide a lighter-weight device that can operate at high or low temperature, has aroused growing research interest. Therefore, the development of a SiC-based hydrogen sensor is an attractive challenge.

In view of the above-mentioned challenges and recent achievements, SiC has been selected for investigation as a H_2_ based sensor in the present work. Herein, Pd/Ta_2_O_5_/SiC and Pd/Ta_2_O_5_/Si Schottky diode type hydrogen sensors are fabricated by radio frequency (RF) sputtering and thermal annealing, and their dynamic response times are observed under various H_2_ gas concentrations and at various temperatures (300, 500, and 700 °C). The results indicate an optimum operating temperature of 300 °C and, under this optimum condition, the Ta_2_O_5_/SiC-based sensor performs exceptionally well, showing excellent sensitivity along with fast rise and fall times of 7 s and 18 s, respectively. These results are significantly better than those previously reported for H_2_ sensors [35,36] and suggest that the Pd/Ta_2_O_5_/SiC-based Schottky diode is a promising candidate for H_2_ sensing applications. To the best of the present authors’ knowledge, few reports on a Pd/Ta_2_O_5_/SiC-based Schottky diode aimed specifically at H_2_ gas sensing have been published [37,38,39].

## 2. Experimental

### 2.1. Material Synthesis and Device Fabrication

**Figure 1:**
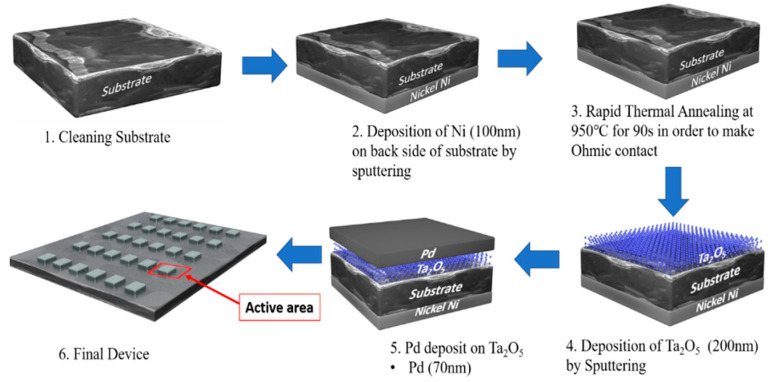
Fabrication procedure for the Pd/Ta_2_O_5_/SiC H_2_ sensor device.

Impurities were ultrasonically removed from the polished and unpolished sides of the substrates by immersing them sequentially in acetone, methanol, isopropyl alcohol, and deionized (DI) water for 5 min each. Thin layers of Ta_2_O_5_ were deposited on the silicon (Si) and silicon carbide (SiC) substrates via radio frequency (RF) sputtering. The natural oxide layer on the unpolished surface of the substrates was removed by buffered oxide etching (BOE) for 10 s, then the samples were rinsed with DI water and dried with N_2_. Nickel (Ni; 100 nm) was deposited as a bottom electrode by sputtering for 30 min at a power of 150 W, followed by rapid thermal processing (RTP) at 950 °C to make an ohmic contact. Next, palladium (Pd) was deposited on the Ta_2_O_5_ thin film using a shadow mask, as shown in Figure 1. In this step, a Pd metal electrode was used as a catalyst for the chemisorption of hydrogen onto the surface because Pd is known to have a very high solubility for hydrogen.

### 2.2. Material Characterization

The crystallinity of the thin films was examined by X-ray diffraction (XRD; Ultima IV, Rigaku Corp., Akishima, Tokyo, Japan) at room temperature under Cu-Kα radiation (0.154 nm) at a potential of 40 kV, a current of 40 mA, and a scan rate of 5°/min in the range of 2θ = 10–80°. In addition, X-ray photoelectron spectroscopy (XPS; PHI 5000 Versa Probe, Ulvac-PHI, Kanagawa,Japan) under Al Kα at 25 W and 6.7 × 10^−8^ Pa was used to confirm the chemical composition and binding energy of the Ta_2_O_5_ film. The morphology of the films was examined via atomic force microscopy (AFM; Vecco Dimension 3100, Veeco Probes Co., Santa Barbara, USA), and the thicknesses and elemental profile of the fabricated device were determined by HRTEM and EDS.

### 2.3. Electrical Characterization

The electrical characterization was performed under an atmosphere of hydrogen and argon using a Keithley Instruments Model 4200A-SCS (Tektronix Co., Oregon,USA) current-voltage (I-V) meter parameter analyzer while varying the temperature from 300 to 700 °C. Hydrogen gas was injected along with the carrier gas (Ar) via a mass flow controller (MFC) at various hydrogen concentrations of up to 5000 ppm. As the H_2_ concentration increases from 0 to 5000 ppm, the H_2_ molecules become dissociated into hydrogen atoms at the surface of the Pd catalyst and are subsequently adsorbed onto the Ta_2_O_5_ surface. This lowers the Schottky barrier height (SBH) and increases the forward current under the elevated temperatures of 300, 500, and 700 °C. The sensitivity of a sensor is defined as the ratio of the sensor resistance under the argon environment (R_a_) to the resistance in pure H_2_ gas (R_g_).

## 3. Results and Discussion

### 3.1. Structural Properties

**Figure 2:**
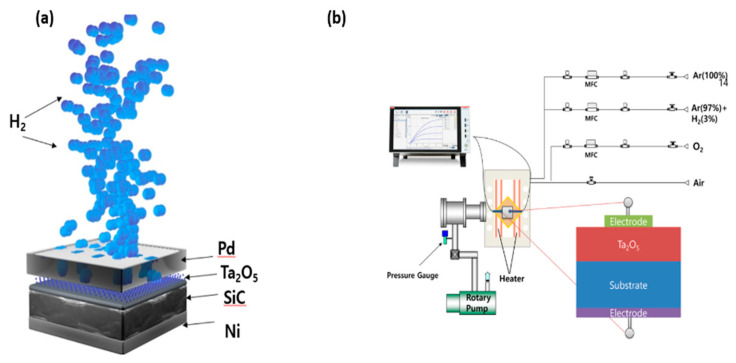
Schematic diagrams of (**a**) the Pd/Ta_2_O_5_/SiC H_2_ sensor, and (**b**) the setup for the electrical measurements.

The Schottky diode hydrogen (H_2_) sensor, consisting of the Ta_2_O_5_ dielectric layer deposited on the SiC substrate, a Pd electrode/catalyst, and an Ni back electrode for ohmic contact, is shown schematically in Figure 2a. In addition, the setup for the electrical measurements is shown schematically in Figure 2b.

**Figure 3:**
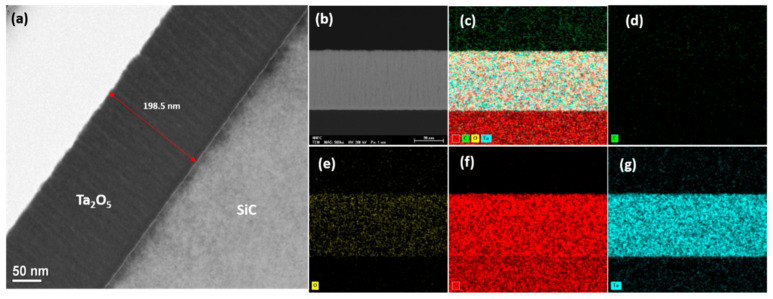
The HRTEM and EDS analysis of the fabricated device: (**a**) a cross-sectional HRTEM image indicating the film thickness; (**b**) the cross-sectional HRTEM image used for the EDS mapping analysis; (**c**–**g**) EDS elemental mappings of: (**c**) combined elements (d)carbon (C), (**e**) oxygen (O), (**f**) silicon (Si), and (**g**) tantalum (Ta).

The sharp interface between the Ta_2_O_5_ (198.5 nm) and SiC is revealed by the TEM image in Figure 3a. The cross-sectional HRTEM image that was used for the EDS elemental mappings of the device is presented in Figure 3b, and the mappings are presented in Figure 3c–g. The corresponding EDS spectrum is provided in Figure S1a of the Supporting Information. The presence of the elements Si, C, O, and Ta provides clear evidence of the Ta_2_O_5_ film on the SiC substrate.

The XRD analysis in Figure S1b clearly reveals the (001), (110), (111), (002), (020), and (200) lattice planes of the Ta_2_O_5_ orthorhombic crystalline phase, thus confirming its formation due to the annealing process at 700 °C under an Ar atmosphere. By contrast, the XRD spectra of the films that were deposited at room temperature (RT), 300 and 500 °C exhibit broad humps with no sharp peaks, thus indicating the amorphous structures of these films.

The AFM images of the Ta_2_O_5_ layers on the SiC and Si substrates are presented in Figure S2a,b, respectively. Here, the surface of the Ta_2_O_5_ film that was grown on the SiC substrate is seen to be rougher than that grown on the Si substrate. In detail, the average roughness of the Ta_2_O_5_ layer on the SiC and Si substrates is 0.645 and 0.484 nm, respectively.

The Ta 4f XPS spectra of the Ta_2_O_5_ thin film deposited on the SiC and Si substrates are presented in Figure S1c, and the corresponding O (1s) spectra are presented in Figure S1d. For both samples, the two peaks located at the binding energies of 26.42 and 28.31 eV in the Ta (4f) spectra are respectively assigned to the Ta (4f_7/2_) and Ta (4f_5/2_) core levels of the Ta^5+^ cations, while the sharp peak located at 531.62 eV in the O (1 s) spectra is associated with the TaO chemical bond [40].

### 3.2. H_2_ Sensor Performances

As the operating temperature is a crucial parameter for gas sensing devices, the electrical and H_2_ gas sensing performances of the Pd/Ta_2_O_5_ Schottky diodes on the SiC and Si substrates were examined under the range of conditions described in Section 2.3. The results in Figure S3 indicate significant response values for both devices at a temperature of 300 °C, whereas poor responses are obtained at lower temperatures due to the slower chemical reaction between the adsorbed H_2_ molecules and the film surface. Moreover, at operating temperatures much higher than 300 °C, the absorbed gas molecules can escape before reaction can occur, so that a poor response is again observed illustrated in Figure S4. These results indicate that the optimum operating temperature for application of the Pd/Ta_2_O_5_ Schottky diode to H_2_ sensing is ~300 °C.

**Figure 4:**
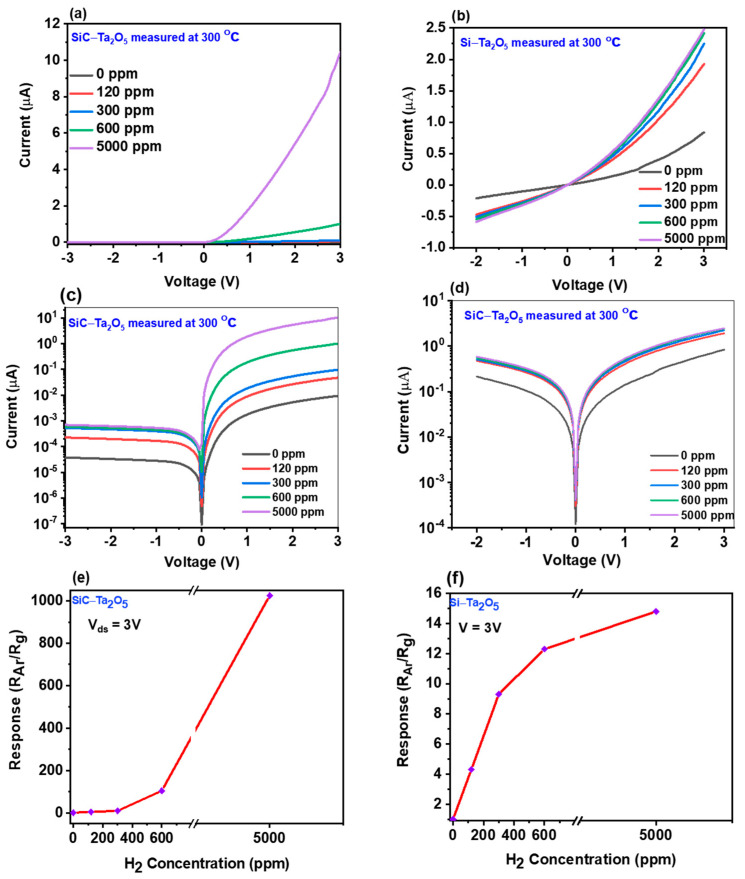
The rectifying diode characteristics of the Pd/Ta_2_O_5_ Schottky diodes as H_2_ sensors: (**a**) and (**b**) the typical linear I-V curves of the device on (**a**) the SiC substrate, and (**b**) the Si substrate, in the presence and absence of H_2_ gas; (**c**) and (**d**) the corresponding logarithmic I-V curves; (**e**) and (**f**) the corresponding responses (R_a_/R_g_) of the sensors as a function of H_2_ concentration.

The rectifying diode characteristics of the Si- and SiC-Pd/Ta_2_O_5_ Schottky diodes in the presence and absence of H_2_ gas at 300 °C are presented in Figure 4. The typical linear I-V curves of the SiC- and Si-based devices are presented in Figure 4a,b, respectively, and the corresponding logarithmic plots are presented in Figure 4c,d. Thus, at a fixed voltage, a significant increase in the forward current is observed as the concentration of H_2_ gas is increased from 0 to 5000 ppm. This is explained by the lowering of the SBH due to the dissociation of the H_2_ molecules into individual hydrogen atoms at the surface of the Pd catalyst, and their subsequent adsorption onto the Ta_2_O_5_ surface. However, the results in Figure 4 also indicate that the sensor that was fabricated on the SiC substrate displays a much higher response than that of the device on the Si substrate.

In addition, the sensitivities of the SiC- and Si-based sensors towards various concentrations of H_2_ gas at the optimum operating temperature of 300 °C are presented in Figure 4e,f, respectively. From this analysis, the resistances of each device at various H_2_ concentrations were extracted and the ratio of the sensor resistance under the argon environment (R_a_) to the resistance in pure H_2_ gas (R_g_) was considered as the sensitivity of the sensor. The results indicated that the SiC-based Pd/Ta_2_O_5_ Schottky diode is much more sensitive than the Si-based device towards H_2_ gas at high operating temperature.

These results can be explained by the large bandgap of the SiC sensor (4.4 eV) [39], which is responsible for minimizing the probability of electron-hole recombination at the Schottky diode junction and, thus, enabling the sensor to function with great stability under harsh temperature conditions. In addition, the higher roughness of the Ta_2_O_5_ thin film on the SiC substrate (as revealed by the AFM image) may further assist the adsorption of H_2_ atoms at the Ta_2_O_5_ surface.

**Figure 5:**
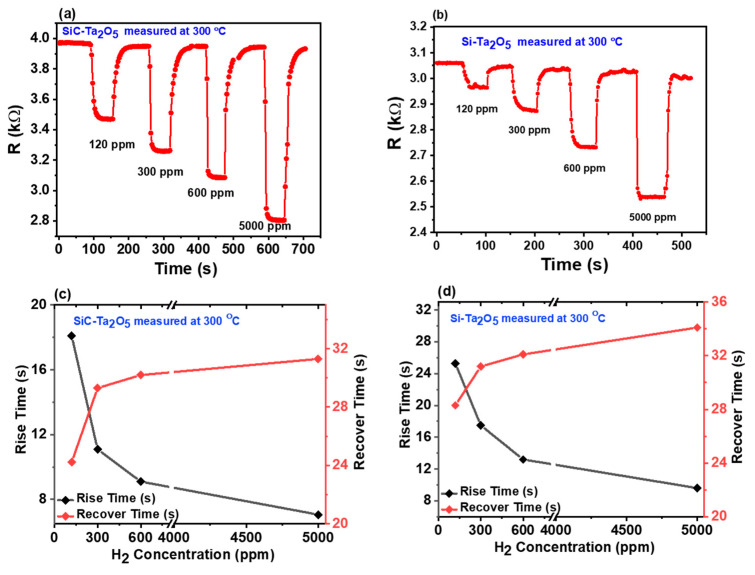
The time-dependent behavior (response and recovery time) of (**a**) the Pd/Ta_2_O_5_/SiC device and (**b**) the Pd/Ta_2_O_5_/SiC device under various H_2_ concentrations at 300 °C. Rising and recovery time Vs H_2_ concentration for (c) Pd/Ta_2_O_5_/SiC device and (d) the Pd/Ta_2_O_5_/Si device

Finally, the dynamic responses of the sensors towards various concentrations of hydrogen gas under a constant bias current at 300 °C are indicated in Figure 5. Here, the SiC sensor exhibits a rapid rise in dynamic response from 10% to 90% in 7.1 s, and a decay from 90% to 10% in 18 s. These results clearly indicate that the SiC sensor has a higher sensitivity towards H_2_ than does the Si-based sensor. Moreover, the results indicate that the response and recovery times of the sensor are highly dependent on the hydrogen concentration.

## 4. Conclusions

In summary, Pd/Ta_2_O_5_ Schottky diodes were fabricated on SiC and Si substrates via radio frequency (RF) magnetron sputtering, and their comparative H_2_ sensing performances were examined in detail under various high temperature conditions. Characterization by atomic force microscopy (AFM) revealed that the roughness of the deposited Ta_2_O_5_ film is higher on SiC than on Si. In addition, the resistance of each device was measured at various concentrations of H_2_ gas. The results revealed that the Pd/Ta_2_O_5_ Schottky diode on the SiC substrate is more sensitive than that on the Si substrate. Moreover, the transient responses of the sensors towards various concentrations of H_2_ gas were investigated, and the SiC-based device exhibited a fast rise time of 7.1 s along with a decay time of 18 s. These results clearly indicate that the sensor based on the SiC substrate has a higher sensitivity towards H_2_ than that based on the Si substrate. The results further suggest that the Pd/Ta_2_O_5_/SiC-based Schottky diode is a rapidly responsive and highly sensitive device for H_2_ sensing under harsh temperature conditions, thus making it a promising candidate for H_2_ sensing applications.

## Data Availability

No new data were created or analyzed in this study. Data sharing is not applicable to this article.

## Supplementary Materials

The following are available online at https://www.mdpi.com/1424-8220/21/4/1042/s1.
