# Supplementary material for: Highly Fast Response of Pd/Ta2O5/SiC and Pd/Ta2O5/Si Schottky Diode-Based Hydrogen Sensors
Source: Sensors (Basel). 2021 Feb 3;21(4):1042. doi: 10.3390/s21041042 (PMC7913499; doi:10.3390/s21041042)
Supplement: Supplementary file 1 [file sensors-21-01042-s001.pdf]

sensors
MDPI

Supplemantary

Highly Fast Response of Pd/Ta2O5/SiC and Pd/Ta2O5/Si Schottky Diode-Based Hydrogen Sensors

Muhammad Hussain [1,†], Woonyoung Jeong [1,†], Il-Suk Kang [2], Kyeong-Keun Choi [3], Syed Hassan Abbas Jaffery [1], Asif Ali [1], Tassawar Hussain [1], Muhammad Ayaz [4], Sajjad Hussain [1] and Jongwan Jung [1,*]

Supporting Information

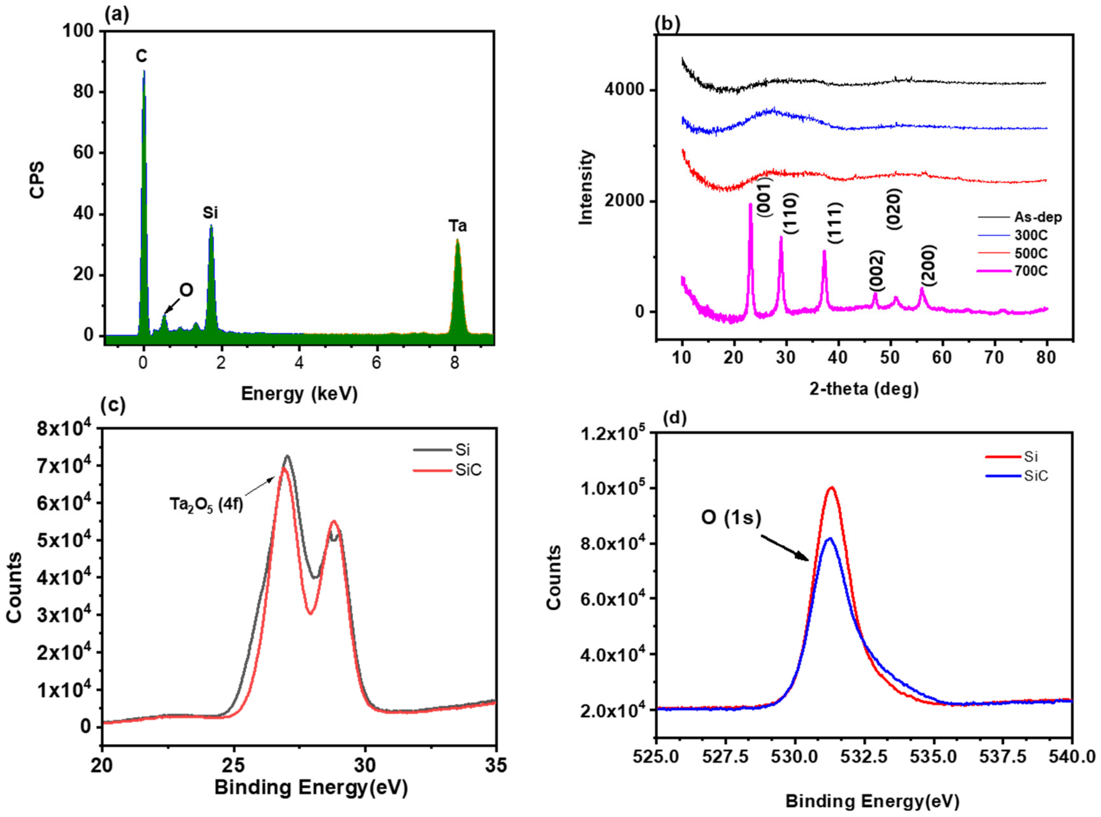

Figure S1. (a) EDS analysis of Ta_2O_5 /SiC sensor (b) XRD analysis of Ta_2O_5 deposited on SiC substrate at different annealing temperature. XPS spectra of binding energy peaks of (c) Ta(4f) and (d) O(1s) deposited on Si and SiC substrates.

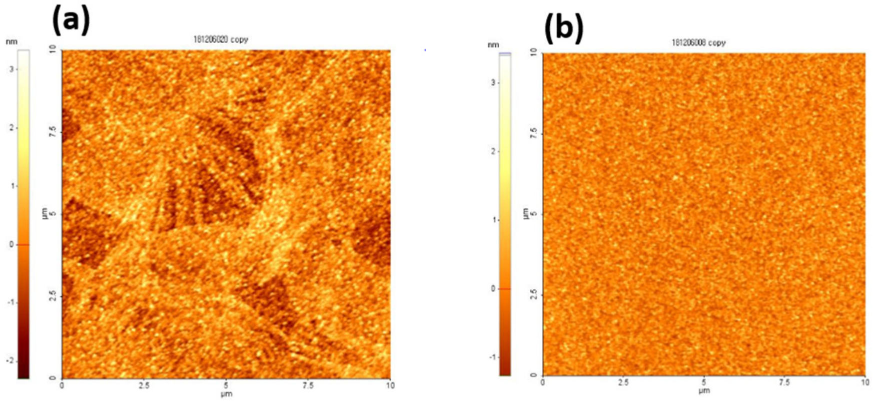

Figure S2. AFM micrographs of **(a)** Ta_2O_5 deposited on SiC substrate **(b)** Ta_2O_5 deposited on Si substrate.

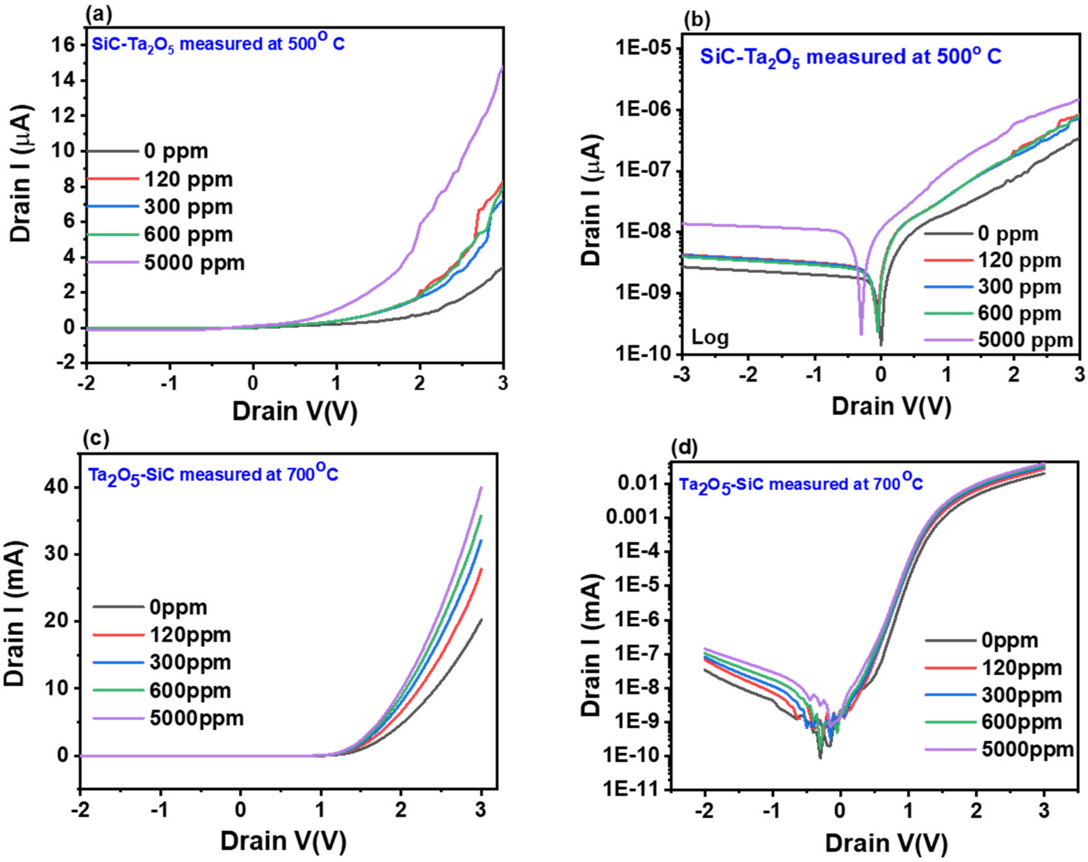

Figure S3. Typical I-V linear curves of Pd/Ta_2O_5 sensors with corresponding logarithmic graph **(a&b)** at 500°C **(c&d)** measured at 700°C.

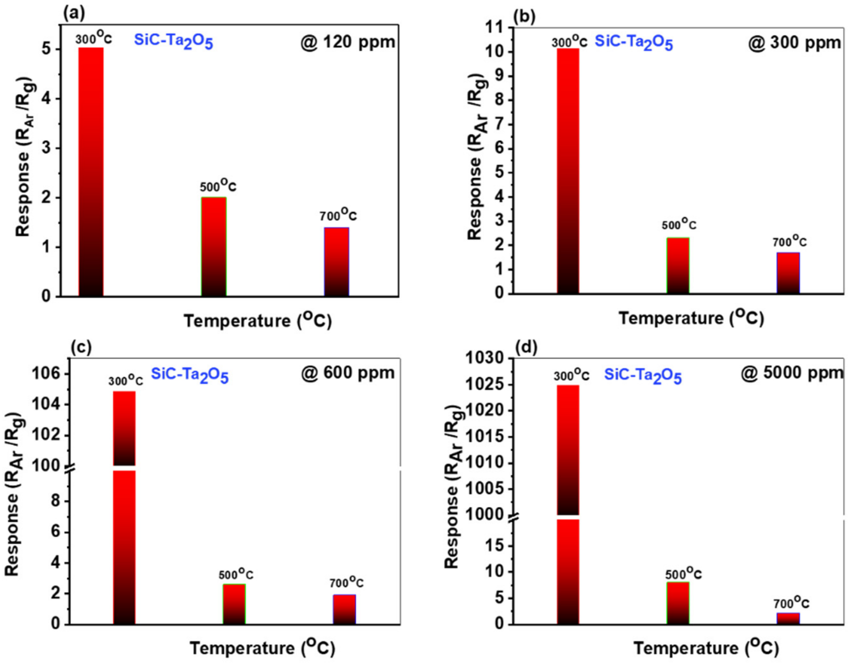

Figure S4. Dependence of the sensor response values on temperature at various H_2 concentrations, **(a)** 120 ppm **(b)** 300 ppm **(c)** 600 ppm and **(d)** 5000 ppm.
